# How to construct liquid-crystal spectacles to control vision of real-world objects and environments

**DOI:** 10.3758/s13428-023-02059-8

**Published:** 2023-02-03

**Authors:** Michael A. Gomez, Jacqueline C. Snow

**Affiliations:** 1 Department of Psychology, The University of Nevada, Reno, 1664 N. Virginia Street, Reno, NV, USA; 2 Psychology Department, Clovis Community College, 10309, N. Willow Ave, Fresno, CA, USA

**Keywords:** Liquid-crystal glasses, Occlusion, Real objects, PLATO visual occlusion spectacles

## Abstract

A major challenge in studying naturalistic vision lies in controlling stimulus and scene viewing time. This is especially the case for studies using real-world objects as stimuli (rather than computerized images) because real objects cannot be “onset” and “offset” in the same way that images can be. Since the late 1980s, one solution to this problem has been to have the observer wear electro-optic spectacles with computer-controlled liquid-crystal lenses that switch between transparent (“open”) and translucent (“closed”) states. Unfortunately, the commercially available glasses (PLATO Visual Occlusion Spectacles) command a high price tag, the hardware is fragile, and the glasses cannot be customized. This led us to explore how to manufacture liquid-crystal occlusion glasses in our own laboratory. Here, we share the products of our work by providing step-by-step instructions for researchers to design, build, operate, and test liquid-crystal glasses for use in experimental contexts. The glasses can be assembled with minimal technical knowledge using readily available components, and they can be customized for different populations and applications. The glasses are robust, and they can be produced at a fraction of the cost of commercial alternatives. Tests of reliability and temporal accuracy show that the performance of our laboratory prototype was comparable to that of the PLATO glasses. We discuss the results of our work with respect to implications for promoting rigor and reproducibility, potential use cases, comparisons with other liquid-crystal shutter glasses, and how users can find information regarding future updates and developments.

## Introduction

Research in psychology and cognitive neuroscience ultimately aims to understand behavior and brain function in response to real-world stimuli and contexts. However, to date, researchers have studied responses to artificial stimuli, typically in the form of pictures or computerized images, rather than real-world exemplars. Images have been favored over real-world stimuli across a range of research domains, including behavioral psychophysics and neuropsychological patient studies, as well electroencephalography EEG and functional magnetic resonance imaging (fMRI). Results from studies of image vision have, in turn, laid the foundation for current theoretical frameworks of human vision, cognition, and action, and the neural systems that support them. The underlying assumption has been that artificial stimuli are adequate experimental proxies for realism for understanding behavior and brain function. However, there is increasing evidence that artificial stimuli drive behavior and brain responses differently than do real-world stimuli. This has led to the realization that realism is critical for understanding human brain function. Yet a major practical hurdle that continues to impede research with real-world stimuli is that it is difficult to present real objects under controlled viewing conditions.

One of the best and most widely used methods of controlling stimulus visibility is to use electro-optic spectacles. The spectacles are composed of a liquid-crystal optical switching material, mounted within the left- and right-eye lenses of a glasses frame. The spectacles that have been used in most experimental contexts since the 1980s are marketed by Translucent Technologies (http://www.translucent.ca/products/plato-visual-occlusion-spectacles/) as “Portable Liquid- crystal Apparatus for Tachistoscope via visual Occlusion” (PLATO) glasses. However, after 15 years of experience using the PLATO glasses, the numerous pitfalls and limitations that we have encountered with the technology ultimately led us to explore how researchers can make their own liquid-crystal spectacles.

In what follows, we first explain why the commercial glasses are unrealistic for many laboratory budgets, and how they are unsuitable for some participant populations and experimental applications. Next, we describe methods that we have developed for researchers to construct their own liquid-crystal spectacles to control a participant’s vision of real-world stimuli. We provide step-by-step instructions to enable researchers to assemble customizable liquid-crystal glasses in their own laboratories. The components of our laboratory prototype are relatively easy to source, and the glasses cost a fraction of that of the commercial alternative. Finally, we conducted performance tests of our liquid-crystal glasses and compared them with the PLATO glasses.

## Methods of controlling stimulus and scene visibility in studies of naturalistic vision

There is clearly a need to expand naturalism in psychology and neuroscience research. Yet there are significant practical challenges to using real objects in laboratory contexts (Romero & Snow, 2019). For (most) studies that try to isolate stimulus and/or task features in the design, one of the biggest challenges is to control the visibility of real objects from the participant’s perspective. Real objects cannot be “onset” and “offset” the same way that computer images can be. The scene in which the objects are presented must also be controlled so that the participant cannot see the experimenter placing the objects on each trial or the items that will be presented on upcoming trials. Real objects are typically presented manually on each trial by the experimenter; this is done either by positioning objects within the experimental scene by hand (e.g., Gerhard et al., 2016; Holler et al., 2019; Snow et al., 2014) or by rotating objects into the scene using a custom-built device, such as a turntable, that holds multiple objects at a time (e.g., Marini et al., 2019; Romero et al., 2018; Romero & Snow, 2019).

A variety of methods have been used to control the visibility of real objects and the experimental scene in laboratory experiments. Although there has been increasing interest in using unconstrained stimuli to understand the “freely-behaving” brain (e.g., Hasson et al., 2004; Maguire, 2012), most studies are designed to control the observer’s vision of the stimuli. Some researchers exert no control over trial timing (e.g., Bushong et al., 2010), which is reasonable in studies where practical constraints place limits on experiments that are already very complex (e.g., Prichard et al., 2021). Others have instructed the participant to open and close their eyes (Herbort & Butz, 2011; Jakobson & Goodale, 1991; Servos et al., 1998), or to look at, or turn away from, the experimental display (Davoli et al., 2011) at moments during the trial. Some studies have controlled stimulus visibility by positioning a barrier or curtain between the participant and the stimulus in between trials (Gerhard et al., 2016; Mischel & Ebbesen, 1970; Snow et al., 2014). However, the temporal precision of these methods is relatively low because they depend on the extent to which participants follow the experimenter’s instructions, or how quickly the experimenter can manually position the occluder. Other studies have controlled visibility of the stimulus using ambient illumination. Using this approach, the testing is conducted in a light-sealed room and the stimulus is illuminated on each trial using a computer-controlled light source, such as a super-bright white light-emitting diode (LED) (Guo et al., 2021). This approach has frequently been used in fMRI studies in humans where the stimuli and equipment are positioned in front of the observer to enable direct viewing of the objects (without a mirror) (Culham et al., 2003; Fabbri et al., 2016; Kroliczak et al., 2007; Kroliczak et al., 2008; Snow et al., 2011), as well as in neurophysiology studies in nonhuman primates (Murata et al., 2000; Sakata et al., 1995; Schaffelhofer & Scherberger, 2016). The approach of using lighting to control stimulus presentation has distinct advantages over the methods described earlier because event timing can be controlled, and participants are not able to see stimulus transitions in between events. However, in our experience, the strong visual transients (dark-to-light transitions) can sometimes be tiring for observers, and the transients create a strong visual response that can complicate contrasts of activation between conditions (i.e., “stimulus” versus “rest,” or “stimulus” versus “fixation” events). Furthermore, testing for prolonged periods in a dark testing environment can lead to dark adaptation between events, which can cause participants to become sleepy.

In the 1980s, human factors research paved the way for a new approach to visual occlusion (Milgram, 1987). The approach consisted of having the observer wear a pair of glasses with a liquid-crystal tachistoscope mounted within the left- and right-eye lenses of the frame. The lenses can switch, with computer-controlled precision, between a transparent (“open”) state, and a translucent (“closed”) state. The technology of the lenses is based on the re-orientation of a liquid-crystal material in response to an applied electrical field, which results in a change in the optical properties of the lens so that incident light is either transmitted (“open”) or scattered (“closed”) (Soni et al., 2013). The lenses are reported to be able to switch between open/closed states within approximately 3 msec (Milgram, 1987), and unlike earlier models with mechanical shutters (Blum, 1957; Ross, 1946), liquid-crystal lenses have the advantage that the switch between states is silent. The technology was subsequently developed and marketed by Translucent Technologies Inc. as “PLATO Visual Occlusion Spectacles” (http://www.translucent.ca/products/plato-visual-occlusion-spectacles/). The glasses have a broad range of applications, including studies of visual perception and cognition, reading, motion perception, action kinematics, motor coordination, and sports psychology. The PLATO spectacles have also been adapted for use in high-field MRI environments (Freud et al., 2018; Podrebarac et al., 2014).

Despite the advantages of using liquid-crystal lenses to control stimulus timing, over the 15 years that we have been using the technology, we have found that the PLATO glasses have numerous limitations. From a practical standpoint, perhaps the most prohibitive factor for many users is the cost. The PLATO spectacles have a unit price of US$ 4000–6000, depending on the hardware that is purchased with the glasses. There is only one available model (“P-1”). The frames of the P-1 are “one-size-fits-all,” and the central divider between the left- and right-eye lenses is 3 cm wide. The arms of the glasses, which house the critical electrical components that control the lenses, are inflexible and fragile. This hardware configuration limits the functionality of the glasses in some study populations, and some experimental contexts. For example, individuals with larger head sizes find the glasses to be uncomfortable, if not impossible, to wear (there have been several instances in our laboratory where a participant has inadvertently snapped one arm off the glasses simply by attempting to put them on). Similarly, young children typically find the glasses to be too large to wear on the head, and the width of the nose bridge produces significant loss of the nasal visual field. The hardware of the P-1 model must be adapted for fMRI and EEG studies to allow removal of the arms (so that the glasses can be positioned in front of the observer’s face, without the arms worn on the head) (Fairchild et al., 2021; Freud et al., 2018). The cost of repairing a damaged unit is expensive (>US$ 300) and typically results in considerable downtime in testing, especially if damaged items must be shipped internationally for repairs.

### A method for constructing and testing liquid-crystal spectacles

Given the drawbacks of the commercially available glasses, we investigated how to build, operate, and test liquid-crystal tachistoscope glasses in our own laboratory. Here, we share the products of our work with a view to supporting open science practices, expanding realism in psychology and neuroscience, and improving rigor and reproducibility in studies of naturalistic vision. Below, we provide step-by-step instructions for sourcing hardware components and assembling liquid-crystal glasses in the laboratory. We also share computer software that we have developed to enable users to operate the glasses and to integrate them with other laboratory equipment. Our liquid-crystal glasses have been tested on computers running both Windows and macOS operating systems. Importantly, we tested the reliability and temporal accuracy of our laboratory-built spectacles, and we compared the performance measures with those of two pairs of PLATO glasses. The results demonstrate that our lab-built prototype was comparable to the PLATO glasses. Importantly, our methods allow for the hardware of the glasses to be customized, for example, to accommodate different sizes and shapes, or for use as a single screen (or “barrier”) rather than wearable glasses. And at ~1/60th of the unit cost of the commercially available alternative, our spectacles are significantly more cost-effective to produce, and if necessary, to repair.

### Tutorial

#### Hardware

The liquid-crystal glasses hardware consists of four major components: a microcontroller, a power switch tail, the vision-occluding material, and the frames. Each component is described in detail below.

#### Microcontroller

The microcontroller is an electronic input/output (I/O) board that receives commands from a computer and uses that information to modulate the state of an output device. The simplest example is sending an instruction from your computer to the microcontroller board which then illuminates an LED light (the inverse relationship is also true: a microcontroller can receive information from an input device [e.g., motion sensor], and send it to the computer). Although a number of microcontroller systems are available, we recommend the Arduino Uno because of its moderate price, reliability, and the excellent technical support available from a large online community of enthusiasts. The system requirements for operating an Arduino Uno are exceeded by most modern computers (e.g., Pentium II, 128 MB RAM, 600 MB hard drive space). Therefore, the remainder of this report describes installing, configuring, and programming using an Arduino Uno microcontroller. We recommend purchasing the Arduino microcontroller directly from the company website (https://store.arduino.cc/usa/arduino-uno-rev3) to avoid the potential risks associated with counterfeit devices.

The Arduino Uno receives coded commands from the experimental computer via a conventional universal serial bus (USB) cable connection. The USB cable must be purchased separately. The commands that the Arduino receives are used to trigger input/output ports (often called “pins”) located on the Arduino. The physical state of electronic devices connected to the pins can then be controlled and modulated. We recommend that the Arduino Uno be housed in a plastic enclosure to decrease the risk of damage to the device due to static discharge or exposure to the environment (an example of a commercially available enclosure: https://www.amazon.com/Enclosure-Transparent-Computer-Compatible-Arduino/dp/B00HFSWC06).

#### Power switch tail

**Fig. 1:**
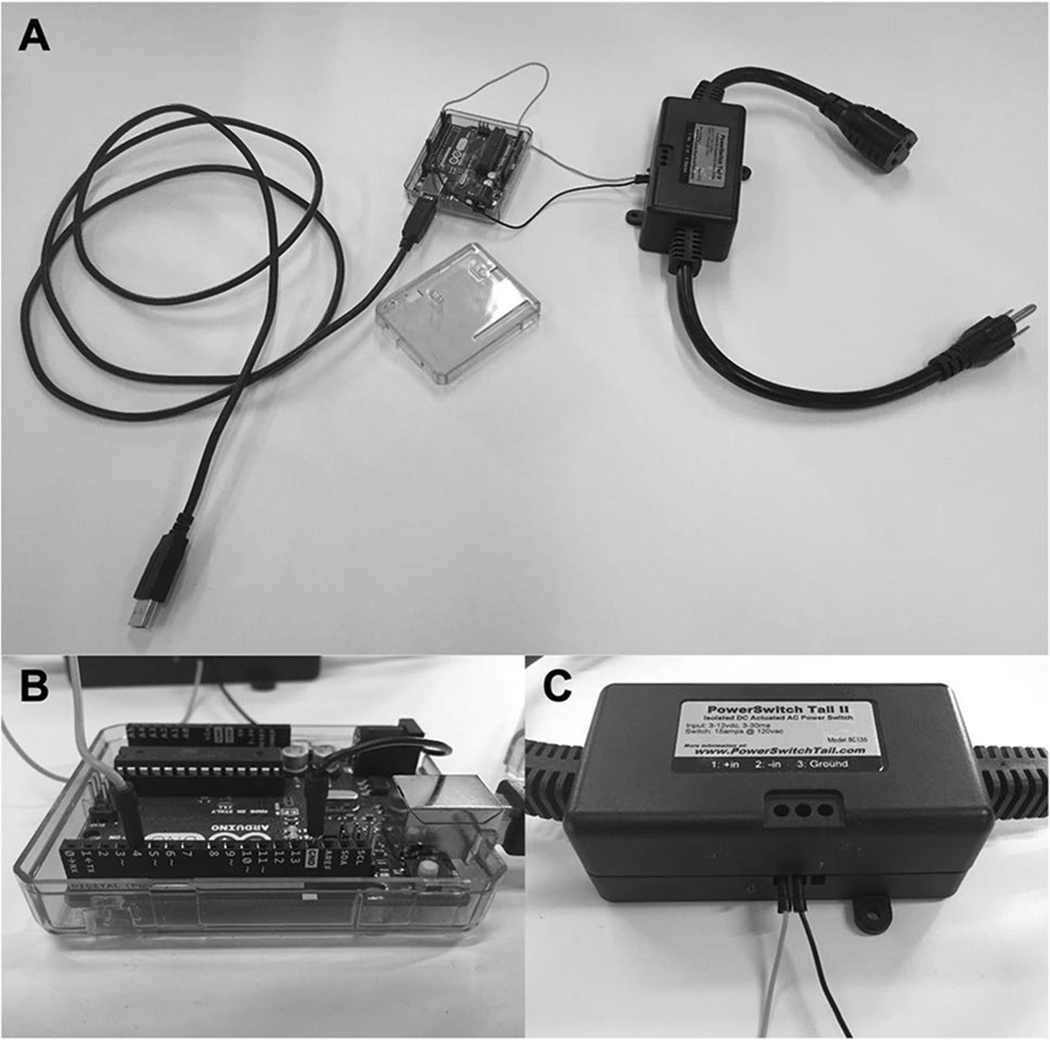
The micro-controller and the power switch tail. (A) The Arduino Uno micro-controller (left) has a USB cable for communication with the experimental computer. This allows the computer to send precisely timed commands to the Arduino at predefined intervals. The power switch tail (right) has an electrical plug on one end and a power outlet on the other. The plug will be connected to an external source of power, and the electrical device to be controlled (e.g., liquid-crystal visor material) will be connected to the power outlet. Jumper wires (signal = light gray, ground = dark gray) connect pin ports on the Arduino Uno (B) to screw terminal ports on the power switch tail (C). This allows the microcontroller to modulate the flow of electrical current from one end of the power switch tail (wall outlet) to the other (vision-occluding material).

**Fig. 2:**
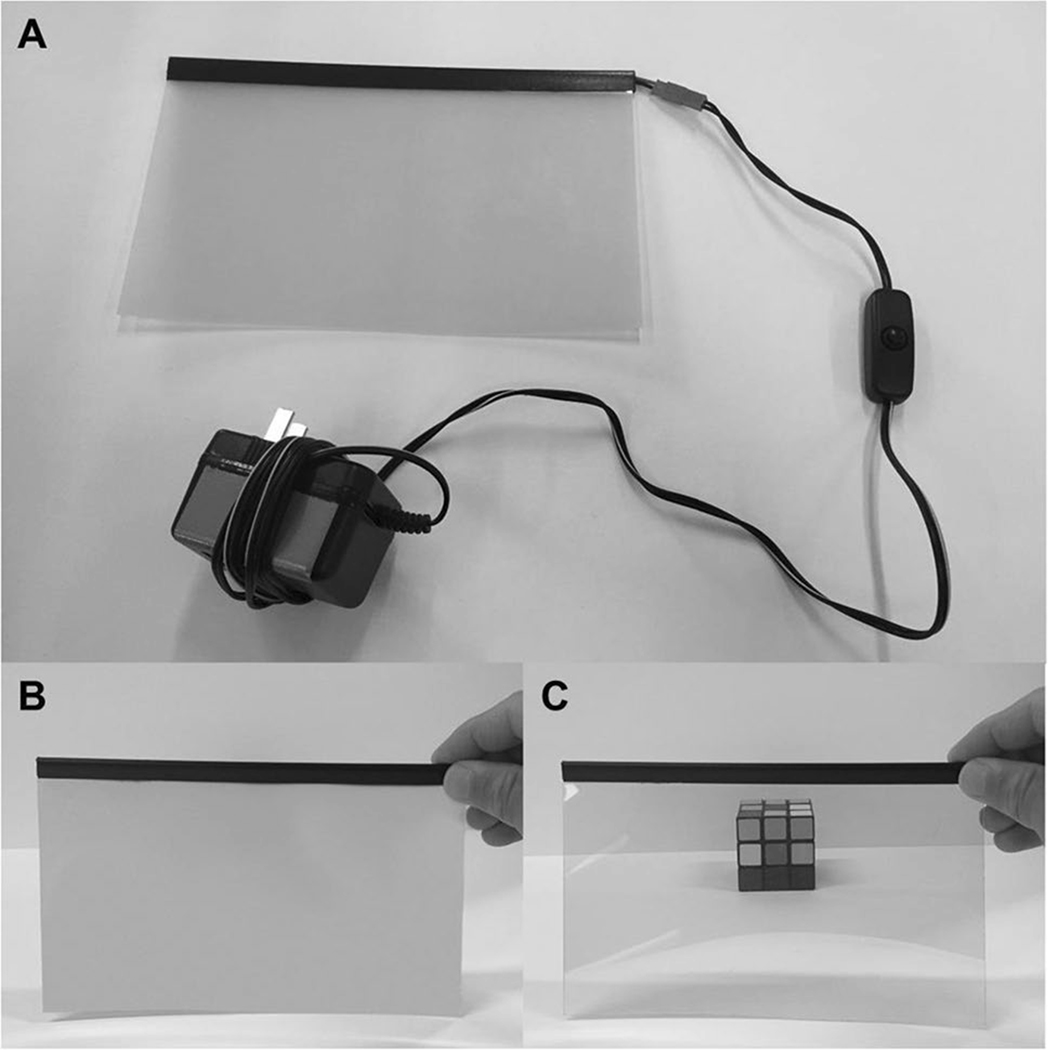
The liquid-crystal lens material. (A) The liquid-crystal material receives power via an AC power supply, which con- nects to the outlet of the power switch tail. The material can also be plugged directly into a wall outlet and modulated from opaque to transparent manually by pressing the on/off button. By controlling the flow of electrical current, the material can switch between translucent (“closed”) (shown in B), and transparent (“open”) (shown in C), states.

The Arduino Uno microcontroller is limited in that it can only modulate the physical state of hardware components that require relatively low voltages to function (e.g., 5 V), such as an LED light. Therefore, controlling the state of higher-power electrical devices, such as those designed to be powered from a wall outlet, requires a power switch tail (alternatively called a “power relay tail”) (Fig. 1A). The primary benefit of using a power switch tail is that the component is preassembled and so modulation of the wall outlet circuit occurs within a protective casing, which minimizes the risk of electrical shock. Although many different models are available, most power switch tails feature a connector for attaching the device to an electrical wall outlet and at least one built-in power outlet for connecting the electronic device that will be controlled (link to the model we used: https://www.probotix.com/Power-Switch-Tail). The power switch tail will have at least two small screw terminal ports (possibly numbered or designated with a “+” or “−” symbol to denote “signal” and “ground,” respectively). If your power switch tail has more than two terminal ports and/ or the ports are not clearly labeled, refer to the instructions of your unit or contact the manufacturer before powering on the device and proceeding with the tutorial. A jumper wire will need to be inserted into a digital pin connector (e.g., pin 4) of the Arduino at one end (Fig. 1B) and clamped into the “+” terminal port of the power switch tail at the other (Fig. 2C). For a list of the digital pin connectors available on the Arduino Uno, see https://content.arduino.cc/assets/A000066-pinout.png. A second jumper wire will need to be inserted into a digital pin connector of the Arduino that is highlighted in white and labeled “GND,” for “ground,” at one end (the Arduino Uno has three ground connectors, and any will do in this case) and inserted and clamped into the “−” terminal port of the power switch tail at the other. Take care to ensure that the signal and ground wires from the power switch tail are connected to the appropriate pin ports of the Arduino, as incorrect wiring could result in damage to the hardware.

We recommend that the electrical work described in this tutorial be completed by a person with proper training to minimize the risk of accidents, such as electrical shocks. The authors shall not be held liable for damages or injuries that are in any way related to, or arise out of, the procedures outlined here.

When a signal from the Arduino pin is sent via the jumper wire to the “+” terminal of the power switch tail, this causes a relay mechanism within the power switch tail to move physically into a position where an electrical circuit is bridged. This bridge allows electrical current to flow from the wall outlet into the connected device (e.g., the visor material). Conversely, when no signal is sent from the Arduino pin, the relay mechanism returns to its default physical state, which breaks the circuit, thus preventing power from reaching the connected device. The power switch tail produces an audible “click” whenever movement of the relay mechanism occurs. If a silent testing environment is important for your design, you might consider using a solid-state relay, which has no moving parts and thus produces no sound during operation. An additional advantage to using a solid-state relay is that the lack of moving parts allows the component to switch states more rapidly than a physical relay. However, due to the increased complexity and precautions involved in safely configuring and operating a solid-state relay, it is beyond the scope of this tutorial and not discussed further here. Playing white noise, either in the testing room or directly to the participant via headphones, is sufficient to mask the “click” emitted by the power switch tail.

#### Vision-occluding material

The third hardware component is the vision-occluding liquid-crystal lens material that will control stimulus visibility. The material is composed of two layers of transparent, electrically conductive material with a liquid-crystal layer embedded in between (Fig. 2A). The chemistry, physics, and mechanics of liquid-crystal materials is beyond the scope of this work (detailed descriptions of these substances and their practical application in perceptual research can be found in Ferguson (1964), Li (2012, Ch. 16, p. 505–522), and Mollon et al. (1977). Briefly, when no electrical current is present, the liquid crystals in the center layer orient randomly; this causes incident light to be scattered by the material, thus making it translucent (Fig. 2B). When a sufficiently high electrical current is applied across the outer layers, the liquid crystals in the center layer align uniformly along the applied field; this allows light to be transmitted through the material, thus making it transparent (Fig. 2C).

After testing liquid-crystal materials from several different vendors, we selected a sample that performed well on our timing tests (link: https://www.amazon.com/gp/product/B01EMYH4JY). It is worth noting that not all vision-occluding materials are suitable for precise timing; in our tests, performance varies widely across brands. We, therefore, recommend that researchers conduct their own performance tests to check how fast and reliable the material is that is used. We also recommend checking the performance of store-bought models. Samples with relatively small dimensions (21 × 29.7 cm) are available. An AC power supply (input: 110 VAC, output: 60 VAC, frequency: 50/60 Hz) that can be connected to any Type A (US, Canada, Mexico, and Japan) electrical wall outlet is included with the material. For other countries, an adaptor must be purchased (refer to the standards and safety guidelines in your region). The material arrives with a semitransparent protective layer on each side, which should be removed before use. One side of the material is slightly adhesive, allowing for it to be affixed to a surface (e.g., transparent acrylic pane) for additional structural support, if necessary. The material can be tested by plugging the power supply into a wall outlet and pressing the on/off button included with the AC power supply cable. The material can be cut with scissors to fit the glasses template (see Lens and frame design). Intricate cuts should be avoided as they can damage the material. The area of the material housing the copper contacts and soldered wire connections should be left intact and uncut at all times. Importantly, never cut the visor material when the unit is connected to, or receiving current from, a power source.

Connect the visor material power supply to the power outlet of the power switch tail. Next, connect the electric connector of the power switch tail to a functioning wall outlet. Finally, press the button on the visor power adaptor wire into the “on” position. The visor material should not change to a transparent state but should still be opaque because the computer has not yet instructed the Arduino to trigger the relay mechanism to complete the circuit and provide power to the material.

#### Lens and frame design

**Fig. 3:**
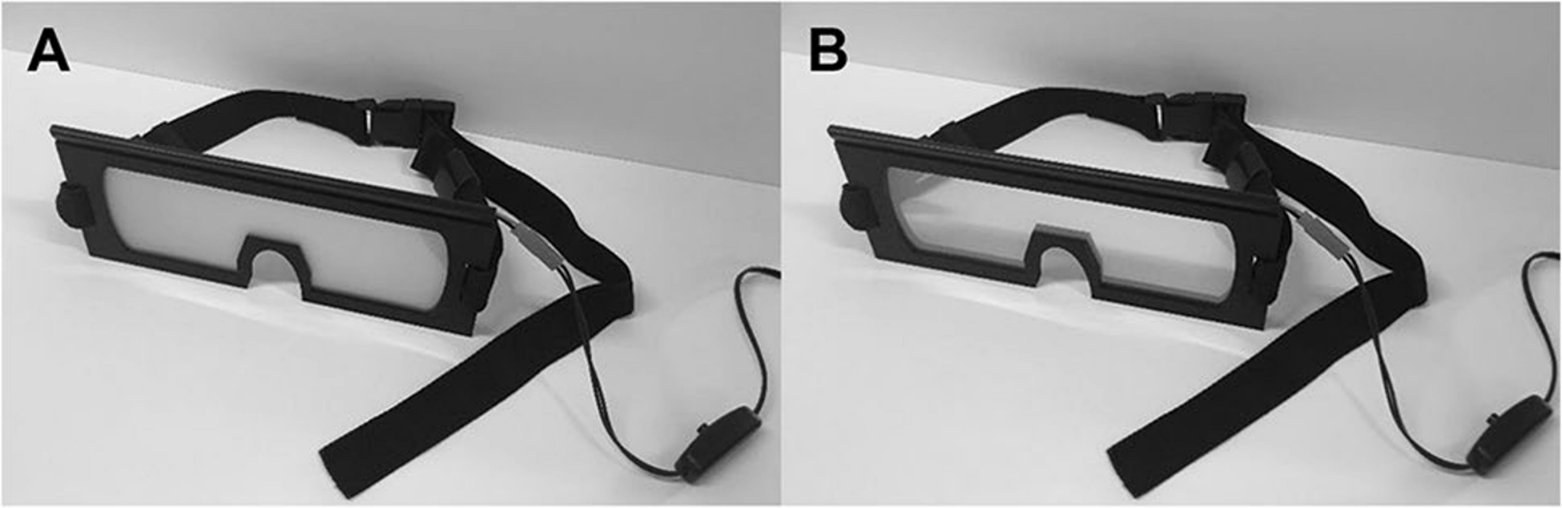
The Laboratory Prototype vision occlusion glasses. Lens mate- rial is shown in (A) translucent and (B) transparent states. The glasses frame was laser-cut from acrylic and medium-density fiberboard. The vision-occluding material was cut to fit within the frame. Adjustable straps are attached to the frames to allow the glasses to be secured around the head of the participant.

The size and shape of the frame that houses the liquid-crystal lens material can be customized to meet the needs of the experimenter (Fig. 3). Below, we provide instructions for making basic glasses frames. Other, more advanced, designs will be accessible on our Liquid-Crystal Glasses Wiki (http://laboratorysys.com/glasses/). We will update the Wiki periodically, but users can also update the Wiki with other designs or with other structural pieces that can be used in combination with the basic frame design.

For example, as we noted above, some researchers use liquid-crystal glasses without handles in fMRI experiments; in this case the glasses can be mounted to the top of the head coil and positioned in front of the participant’s eyes to control the visibility of stimuli mounted in front of the participant. Designs for adjustable mounting devices could also be valuable future additions to the Liquid-Crystal Glasses Wiki page.

To make the basic glasses frames, start by downloading and printing our Lens Template file (laboratorysys.com/glasses/files/Lens_Template.pdf). Cut along the dotted lines of the template to produce the basic frame shape. Also cut along the unbroken line at the top of the template. Next, use tape to temporarily affix the template to the vision-occluding material. Position the template so that the unbroken line is parallel and adjacent to the top of the material where the copper contacts and soldered wire are housed. As mentioned above, the vision-occluding material can be cut using scissors. Before cutting, check that the material is not plugged in or receiving power. Using the dotted lines in the template as a guide, cut the liquid-crystal material to match the template. Do not cut the vision-occluding material along the unbroken line of the template, as this will prevent electrical current from reaching your lens pieces.

For building the frame of the glasses, we recommend using one of two methods. The frame pieces can be laser cut (download our “2-D Frame” file package: http://laboratorysys.com/glasses/files/2D_Frame.pdf). For this method, we recommend using medium-density fiberboard (MDF) that is 1/4″ thick or less. Alternatively, the frame can be designed and 3-D-printed in plastic based on the needs of the researcher.

Modifications of the basic design are straightforward: simply produce your own template for cutting the vision-occluding material, and/or create 3-D files for producing a frame of the appropriate size and shape. For example, the frame could be rectangular, thereby producing a vision-occluding barrier.

#### Software

Although the majority of the software required to run our liquid-crystal glasses is freely available online, the following tutorial describes a hardware setup controlled by the proprietary computing environment and programming language MATLAB (MathWorks). If a MATLAB license is unavailable, the General Public License (GNU) project Octave (not covered further here) provides a programming environment and language comparable to MATLAB that is Windows/ Mac/Linux compatible and is free to download and use (Octave: https://www.gnu.org/software/octave/; Arduino package: https://wiki.octave.org/Arduino_package). As computer hardware and software are updated by manufacturers and programmers, we will endeavor to provide alternatives to some of the components and/or code outlined below on our glasses Wiki page: http://laboratorysys.com/glasses/.

Below, we describe how to send commands from the experimental computer to the Arduino, which in turn modulates the position of the relay mechanism in the power switch tail to impede or allow the flow of electrical current into the vision-occluding material so that it can switch between opaque and transparent states. First, download and install the Arduino support package. This process is the same for Windows and macOS computers. It is assumed that you have followed the steps outlined above and that MATLAB is installed on the experimental computer.

For more recent versions of MATLAB (e.g., 2014a and later), the current support package for the Arduino can be downloaded and installed 1) manually (download link: https://www.mathworks.com/matlabcentral/fileexchange/47522-matlab-support-package-for-arduino-hardware) or 2) automatically from within the MATLAB graphical user interface. To automatically download and install the support package, open MATLAB on your experimental computer and click: Home » Add-Ons » Get Hardware Support Packages » MATLAB Support Package for Arduino Hardware. Follow the prompts to complete installation. Earlier versions of MATLAB (e.g., 2013b and prior) might not support download/installation of the Arduino support package, so the legacy MATLAB support package must be downloaded and installed manually (link: https://www.mathworks.com/matlabcentral/fileexchange/32374-legacy-matlab-and-simulink-support-for-arduino). This requires that the Arduino Integrated Development Environment (IDE), which is a simple graphical user interface for loading scripts directly to the Arduino, also be installed (https://www.arduino.cc/en/Main/Software). In order for MATLAB to control the Arduino using the legacy package, you must first upload a program to the device via the IDE. The program can be found using the following path within the legacy software package: ArduinoIO » pde » adios » adios.pde. Refer to the Arduino site for instructions on loading a program onto the Uno board via the IDE (https://www.arduino.cc/en/Guide/ArduinoUno#toc4). Be aware that the MATLAB code commands used to operate devices connected to the Arduino vary depending on whether the current or legacy software package is used. In addition, the code used to initialize communication between MATLAB and the Arduino varies slightly depending on whether a Windows or macOS computer is used.

#### Computer code

When an Arduino is connected for the first time to a computer running Windows, the computer will assign the Arduino a “COM” port value. This value is necessary for ensuring that the Windows computer can communicate properly with the Arduino. The COM port value can be found by navigating within the Windows OS: Control Panel » System » Device Manager » Ports (COM & LPT). Within the Ports tab, you should see that “Arduino Uno” is present, followed by the word “COM” with an adjacent numerical port value [e.g., “Arduino Uno (COM6)”]. It is important that you make note of this COM port value, as it may be required below for coding the hardware connection. At the top of your experimental program, you need to initialize communication between MATLAB and the Arduino using the following code:


a = arduino(); %Initialize a connection between Matlab and the Arduino


If you are using a hardware setup where multiple Arduinos are connected to the experimental computer simultaneously, use the code below (with the “COM” port value being the one for the Arduino that is wired to the power switch tail, e.g., 6):


a = arduino(‘COM6’,’Uno’); %Initialize a connection between Matlab and the Arduino (multiple Arduinos scenario)


Unlike Windows computers, macOS computers do not automatically assign and manage communication port values for each connected USB device. Instead, communication with the USB ports involves default directory paths that are preset by the operating system. Below is the code if using either the *current* or *legacy* MATLAB support packages. At the top of your experimental program, you need to initialize communication between MATLAB and the Arduino with the following code:


a = arduino(‘/dev/cu.usbmodem1411’,’Uno’); %Initialize a connection between Matlab and the Arduino


Although this approach works for the latest macOS that we tested (Sierra, 10.12.6), it may be that current and/or future updates will render this method obsolete (check our Glasses Wiki for updates: http://laboratorysys.com/glasses/).

Next, we explain the process for switching the state of the vision-occluding material from opaque, to transparent, and back to opaque. Below is the code if using the current MATLAB support package (Windows/Mac). To send a signal via the Arduino to the power switch tail and cause the visor material to become transparent, use the code below (with the numerical value adjacent to “D” being the number of the pin port on the Arduino that is sending a signal to the power switch tail, e.g., 4):


writeDigitalPin(a, ‘D4’, 1); %Transparent


We use the WriteDigitalPin command because we are writing (or “sending”) a command to digital (“D”) pin “4” of the Arduino that is initialized to communicate using variable “a.” The “1” reflects the digital (i.e., binary) nature of the pin and represents a “true,” or “on,” statement. Executing this same command, but replacing the “1” with a “0” (i.e., “false”) value, will cause the material to become opaque again:


writeDigitalPin(a, ‘D4’, 0); %Opaque


Below is the code if using the *legacy* MATLAB support package (Windows/Mac). As with the current support package, you must first initialize communication between MATLAB and the Arduino at the start of your program using the following code:


a = arduino(‘COM6’); %Initialize a connection between Matlab and the Arduino
a. pinMode(4,’output’); %Initialize the pin on the Arduino and its mode


In the first line of code above, it is important that the “COM” port value be the one for the Arduino that is connected to the power switch tail and visor material (e.g., 6). Also, in the second line above the numerical value within the parentheses must be the number of the pin port on the Arduino that is wired to send a signal to the power switch tail (e.g., 4). The material can be switched to a transparent state using the code below:


a.digitalWrite(4,1); %Transparent


We use the “digitalWrite” command because we are writing (or “sending”) a command to pin “4” of the Arduino initialized to communicate using variable “a.” The “1” reflects the digital (i.e., binary) nature of the pin and represents a “true,” or “on,” state. Executing this same command, but replacing the “1” with a “0” (i.e., “false”) value as below, will cause the material to switch back to an opaque state:


a.digitalWrite(4,0); %Opaque


#### Performance tests

Next, we conducted performance tests on our liquid-crystal glasses prototype, and on two sets of PLATO glasses. Testing for all three sets of glasses was conducted using the same testing equipment, apparatus, and procedures, described below. Note that unlike the PLATO glasses, which are designed to allow the left and right lenses to switch independently between “open” and “closed” states, the material for our glasses consists of a single contiguous lens panel.

In principle, our glasses could be constructed with two independent lens panels (like the PLATO glasses) so that they could serve a demand for monocular versus binocular stimulus presentation (which the PLATO glasses do); however, our initial prototype uses a single lens panel because it is easier to construct (see Alternative approaches and applications). A single panel should, in principle, also reduce the likelihood of timing inconsistencies between the left- and right-eye lenses during normal binocular viewing (see Performance test results). In cases where monocular viewing conditions are desired, an eye patch may be used to eliminate visual input from one eye. However, using an eye patch is problematic because it removes the opportunity for randomization of the two viewing conditions. Further patching (typically) results in dark adaptation of the occluded eye. LC shutters may therefore be preferable for this use as the occluded eye receives similar light levels.

#### Test equipment and apparatus

For each timing test, we used a photodiode, which was affixed to one lens (left/right) of the glasses, to detect changes in luminance (converted to Volts). Signal magnitude, which was recorded in real-time, was amplified (Tramp Transimpedance Amplifier, UDT Instruments), with gain select set so that the switch in transparency could be differentiated from background noise (107). Data were acquired using a National Instruments (National Instruments, Newbury, Berks, UK) data acquisition device (sample rate: 1000 Hz). The photodiode was positioned over the approximate location on the inside of the lens where the left or right eye would be during binocular use. Testing was conducted using a Windows 7 PC (Intel i7 3.4 GHz, 16 GB RAM) running MATLAB (R2017b, 64-bit) with the Psychophysics Toolbox extensions (Brainard, 1997; Kleiner et al., 2007; Pelli, 1997). To minimize interference of extraneous background Windows processes on the performance results, we used the Psychtoolbox-3 Priority command (setting = 1, “high priority”). A box (rectangular prism, 31 × 27 × 27 cm) was constructed from a black foam core to house the glasses and photodiode during testing; the box provided a testing environment in which external illumination was controlled. A small USB powered tabletop lamp (IdeaWorks, Super Bright Portable LED Lamp, white) provided a consistent source of illumination inside the container. The lamp was positioned on the side of the lens opposite the photodiode. Small holes were cut into the side of the box to accommodate the wires of the photodiode, glasses, and lamp; black duct tape was affixed around the entry points to the box to prevent external light from infiltrating the box.

#### Test procedures and data analysis

We first tested temporal *onset latency*—the time (msec) taken for the liquid-crystal lens to transition from “closed” (translucent) to “open” (transparent) state. Each trial started with the glasses in a closed state for 500 msec. The glasses were then triggered, using MATLAB, to switch to the “open” state. The glasses were programmed to remain “open” for 500 msec, after which time they were triggered to return to the “closed” state. The trial concluded with a 1000 msec inter-trial interval (ITI). Data were collected during the initial 1000 msec of the trial. Timing data were collected separately for each “lens” (left/right side) of each of the three sets of glasses (laboratory prototype, PLATO 1, PLATO 2), yielding a total of 6000 trials. For our laboratory prototype, we describe the results of tests conducted on both the left and right sides of the single pane since it was important to determine whether onset timing was uniform across the panel.

Next, we tested temporal *offset latency*—the time taken for the liquid-crystal lenses to transition from the “open” to the “closed” state. We used the same procedures as for the onset tests, except that the lenses started in the “open” state for 500 msec, then changed to the “closed” state for 500 msec, and then returned to the “open” state for 1000 msec (ITI).

To calculate onset and offset latency, we first recorded from the photodiode for 5 seconds, both when the vision-occluding material was translucent and when it was transparent, and computed the average signal magnitude in each state. The lens was defined as having reached a transparent or translucent state at the timepoint (msec) when the magnitude of the photodiode signal fell within one standard deviation of the transparent or translucent mean, respectively.

For both the onset and offset latency tests, we measured for each set of glasses (PLATO 1, PLATO 2, laboratory prototype) and for each side (left/right) the standard deviation (SD), the minimum (Min) and maximum (Max) values of the distribution (i.e., the range), the Mode, and the frequency of the mode of the onset latency values. The mean onset and offset latency test data were analyzed using mixed-model analysis of variance (ANOVA) treating glasses (PLATO 1, PLATO 2, and laboratory prototype) as a “between-subjects” factor, and side (left, right) as a “within-subjects” factor. Main effects of glasses were decomposed using independent-samples t-tests. Two-way interactions between glasses and side were examined initially using paired-samples t-tests across left and right sides, separately for each set of glasses. To compare the left- and right-side differences in onset/offset latency directly, we computed a *difference index* by subtracting the mean onset/ offset latency for the right side from the left side (i.e., left side − right side) separately for each set of glasses. We compared the resulting indices using one-way ANOVA and follow-up independent-samples t-tests.

#### Performance test results

##### Onset latency

**Fig. 4:**
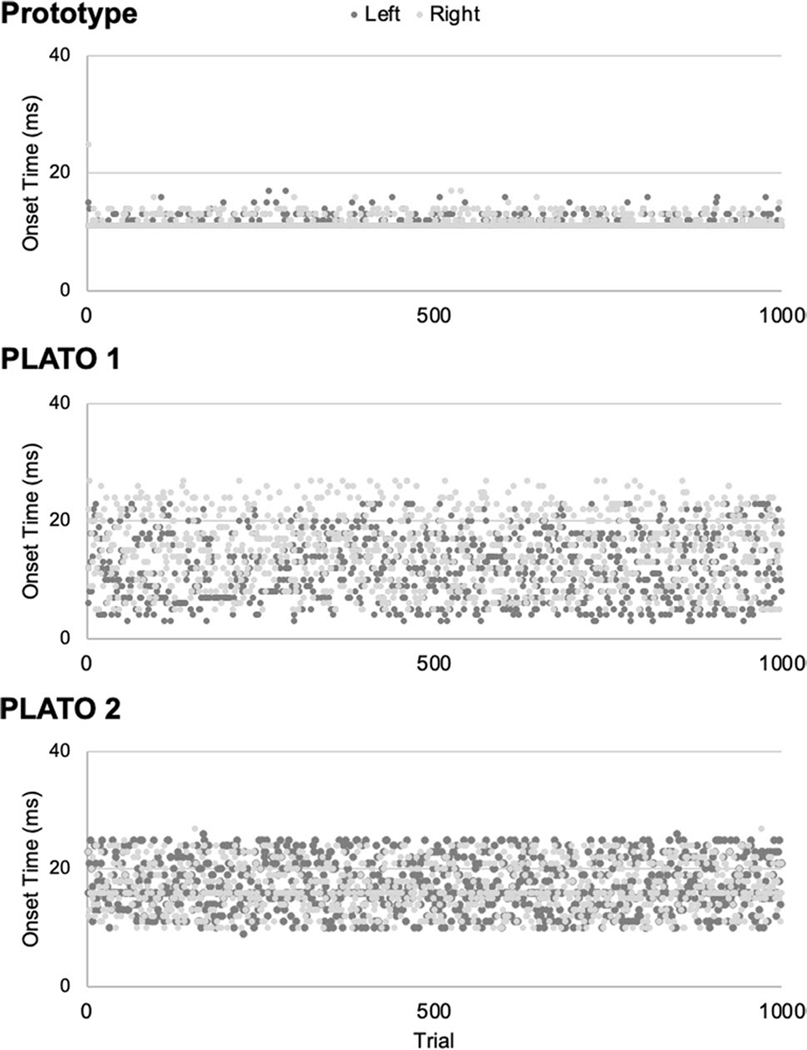
Onset latencies for the laboratory prototype (upper panel), PLATO 1 (middle panel), and PLATO 2 (lower panel) glasses across 1000 test trials. Data are shown separately for each side (left, right). The first 1–2 trials for each lens of each pair of glasses exceeded the upper limit of the y-axis, and so for visualization purposes these trials are not depicted in the figure (see Table 1, Max values).

*Onset latency* was calculated as the time elapsed (in msec) from when the coded instruction to switch states was delivered via MATLAB until the glasses switched from an opaque (“closed”) to translucent (“open”) state. Onset latencies across the 1000 test trials are shown separately for each set of glasses and each side in Fig. 4. Descriptive statistics for the onset latency tests for each set of glasses (PLATO 1, PLATO 2, laboratory prototype) and each side (left/right) are shown in Table 1. The results of the statistical tests of onset latency are summarized in Table 2.

The main outcomes of the onset timing tests are summarized below. Overall, mean onset latencies across all sets of glasses ranged between 11–18 msec (Table 1). While there were differences in onset speed across the three pairs (see Table 2, main effect of glasses), the laboratory prototype performed very well (mean onset time: prototype = 11.49 msec; PLATO 1 = 13.69 msec; PLATO 2 = 17.13 msec). Moreover, as is evident in Fig. 4 and from Table 1, the standard deviation in onset times for the laboratory prototype was comparatively low (prototype = 1.59 msec; PLATO 1 = 5.89 msec; PLATO 2 = 4.43 msec), demonstrating remarkably consistent onset timing across trials. The three sets of glasses also differed in the extent to which onset latencies varied between the left versus right lens sides (Table 1). Specifically, for the PLATO glasses, there were significant differences in onset latencies between the left and right lenses, whereas this was not the case for our prototype (Table 2: glasses × side interaction). This result is perhaps not surprising since our prototype consisted of a single sheet of material which was tested separately on the left and right sides, whereas the PLATO glasses have separately controlled panels for the left versus right lenses. Nevertheless, these data caution that there can be variability in onset latencies across the left and right sides of the glasses when they are controlled separately.

##### Offset latency

**Fig. 5:**
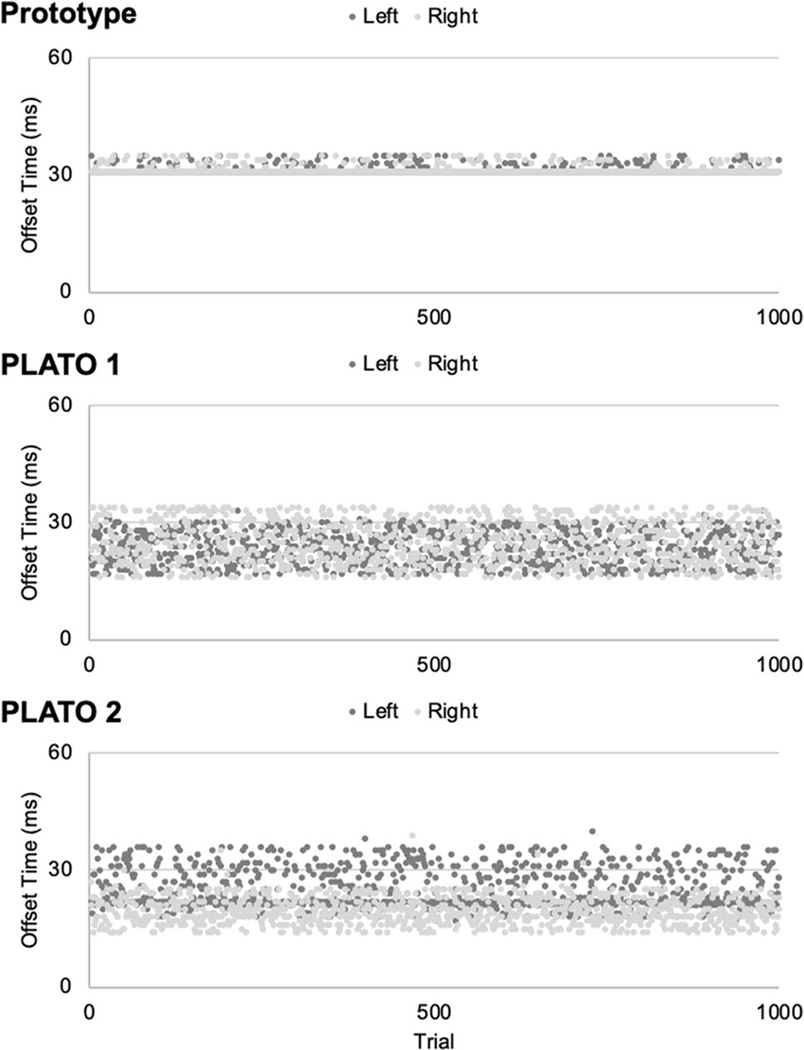
Offset latencies for the PLATO 1 (upper panel), PLATO 2 (middle panel), and laboratory prototype (lower panel) glasses across 1000 test trials. Data are shown separately for each side (left, right). The first 1–2 trials for each lens of each pair of glasses exceeded the upper limit of the y-axis, and so for visualization purposes, these tri- als are not depicted in the figure (see Table 3, Max values).

*Offset latency* was calculated as the time elapsed (in msec) from when the coded instruction to switch states was delivered via MATLAB until the glasses switched from a translucent (“open”) to an opaque (“closed”) state. Offset latencies across the 1000 test trials are shown separately for each set of glasses and each side in Fig. 5. Descriptive statistics for the offset latency tests for each set of glasses (PLATO 1, PLATO 2, laboratory prototype) and each side (left/right) are shown in Table 3. The results of the statistical tests of offset latency are summarized in Table 4.

The main outcomes of the offset timing tests are summarized below. Overall, mean offset latencies across all sets of glasses ranged between 19–32 msec (Table 3). There were differences in the speed of offset across the three pairs of glasses (Table 2, main effect of glasses). Our laboratory prototype had the slowest offset time (prototype = 31.40 msec; PLATO 1 = 24.18 msec; PLATO 2 = 22.39 msec)—a mean offset difference of ~8 msec relative to the PLATO glasses. However, as is evident in Fig. 5 and Table 1, the standard deviation in offset times for our prototype was comparatively low (prototype = 1.68 msec; PLATO 1 = 4.88 msec; PLATO 2 = 4.78 msec). Therefore, although our prototype glasses were slightly slower to offset than the PLATO glasses, their offset timing was strikingly consistent across trials, which is similar to what we observed in the onset tests. Also similar to the onset timing tests, the three sets of glasses differed in offset latencies between the left versus right sides (Table 4, glasses × side interaction). Specifically, for both sets of PLATO glasses, the left and right lenses had significantly different offset latencies (mean difference PLATO 1 = 1.45 msec; PLATO 2 = 5.49 msec), whereas this was not the case for the prototype (mean difference left vs. right side = 0.01 msec). Again, this is likely to reflect that the left and right sides of our prototype consisted of a single sheet of material, unlike the PLATO glasses, for which the left and right sides consist of separate liquid-crystal panels.

## Discussion

A major practical hurdle that continues to impede research studies of naturalistic vision is that it is difficult to display real objects and environments under controlled viewing conditions. Here, we described step-by-step methods for researchers to create, test, and use liquid-crystal visual occlusion glasses. We explained how the spectacles can be assembled, even by users who have minimal technical knowledge, using readily available electronic and hardware components. The basic design that we have used for our liquid-crystal glasses can be customized in size or shape so that the hardware can be worn (e.g., by individuals with different head sizes), or otherwise modified to suit a variety of experimental applications.

### Performance tests and comparisons with a commercial model

We conducted careful timing tests to measure the performance of our laboratory prototype glasses, and we compared our model with two pairs of PLATO glasses (PLATO 1, PLATO 2). We conducted tests of both onset latency—the time taken (msec) for the liquid-crystal lenses to transition from “closed” (translucent) to “open” (transparent) state, and offset latency—the time taken for the lenses to change from “open” to “closed” state.

The results of our timing tests demonstrate that the laboratory prototype performed comparably well in contrast to the PLATO glasses. While there were some minor differences across glasses in the average speed of onsets and offsets, both between the prototype versus the PLATO glasses, and between different sets of PLATO glasses, overall, our tests showed that the prototype performed comparatively well with respect to both speed and consistency. There were differences in the speed of onset and offsets across the left versus right sides of the PLATO glasses, which were not apparent with our prototype. This result is not surprising given that the left and right sides of our prototype consisted of a single sheet of material (unlike the PLATO glasses), but the results underscore how separate lenses can result in variability in both onset and offset times.

Some experimenters may wish to control separately the left- and right-side lenses, for example, to enforce monocular viewing of the stimuli. Although our laboratory prototype could be constructed with two independent lens panels (similar to the PLATO glasses), as we suggested earlier, and as our tests have confirmed, the single panel reduces the likelihood of timing inconsistencies during normal binocular viewing. We recommend careful timing tests for users who wish to extend our model for use with separate left versus right lenses.

### Promoting rigor and reproducibility in naturalistic vision research

Precisely controlling stimulus timing represents a major challenge for previous studies of naturalistic vision. A common assumption is that artificial stimuli (such as computerized images) provide excellent experimental control at the cost of ecological validity, whereas real-world stimuli provide the converse (Snow & Culham, 2021). However, using real-world stimuli does not necessarily mean that control over stimulus parameters must be sacrificed; on the contrary, both control and ecological validity can be maximized with carefully designed and tested apparatus and protocols (Romero & Snow, 2019). Although a variety of methods have been used in previous studies, liquid-crystal glasses provide excellent temporal control over the participant’s vision of the stimuli and the experimental scene. Unlike with experimenter-operated shutters or barriers, or approaches that rely on participants to follow experimenter instructions about when to look at the stimuli, the transition between “open” (visible) and “closed” (not visible) states with liquid-crystal glasses is silent, temporally precise, and under the experimenter’s control.

The methods outlined in this paper strengthen scientific rigor because they provide researchers with information about how to manufacture, test, and repair liquid-crystal vision occlusion glasses in the laboratory, with the aim of maximizing temporal control over stimulus/scene visibility. Controlling stimulus presentation can improve experimental methodology and strengthen the interpretation of results.

We have also provided instructions for constructing custom frames within which to mount the liquid-crystal lenses. Well-fitting frames should improve wearability, reduce participant discomfort, and ultimately, improve data quality. Customized frames also allow studies of naturalistic vision to be more inclusive: studies can be extended more easily to other populations (such as young children) and to other applications (such as in studies requiring screens rather than glasses, or for use in fMRI environments where space constraints can place limits on the optimal size and shape of the glasses).

These methods will be useful both for researchers who want to start to incorporate realism into their research, and for researchers who already use liquid-crystal glasses to control real-world displays, but who find that the available commercial models are limited in design or functionality. Our method should be especially useful for researchers who must operate on limited budgets, or for those who wish to reallocate research funds away from the apparatus for data collection.

### Applications and comparisons with other liquid-crystal shutter glasses

Although we have focused on comparing our liquid-crystal glasses prototype with the PLATO glasses, users are encouraged to research other commercial products that may be available, and that use similar technology. StereoGraphics Corporation (San Rafael, CA) produces head-mounted active shutter glasses called “CrystalEyes” (current retail: ~US$ 1000). The glasses are used in conjunction with an infrared (IR) receiver to synchronize the opening of the left and right lenses with alternating images displayed on a computer monitor (i.e., alternating monocular presentations). That is, the images presented to the left and right eyes on the computer monitor can depict different viewpoints of a stimulus or scene from the perspective of the left and right eyes, respectively, and when the images are presented in rapid alternation, this produces a unitary percept of3-D depth. The CrystalEyes technology is analogous to the glasses/receiver approach used in the nVidia (Santa Clara, CA) GeForce 3D vision kit (current retail: ~US$ 99–150), which is marketed for 3-D video gaming applications, but which has also been used in scientific studies of 3-D image vision (e.g., Gomez, et al. 2018).

How, then, should researchers decide which type of glasses are best to present and control stimulus visibility? The answer to this question likely depends on the use case. Important questions to consider are whether the glasses will be used primarily to present computerized images or real-world solid objects (or other noncomputerized stimuli), and whether there is a need for stereoscopic image presentations that require rapidly alternating inputs to each eye. The CrystalEyes and nVidia systems are designed for computerized image presentations, and the IR receiver ensures rapid alternating monocular images for 3-D stereoscopic vision. These systems could, in principle, be programmed so that both lenses open/close simultaneously, which would make them functional for controlling vision of real-world objects (not just computerized images). However, in our experience, when these glasses are in the “open” state the lenses have a tint that affects the visibility of solid object stimuli.

The PLATO glasses have separately controlled left and right lenses, and therefore may permit 3-D stereoscopic presentations. Unlike the CrystalEyes and nVidia systems, the lenses of the PLATO glasses are not synchronized using an IR sensor, leaving open the possibility of synchronization issues in cases where onset/offset latencies are delayed (see our Performance test results). While there appear to have been some other early tests of this technology for 3-D stereoscopic applications, users are encouraged to perform careful timing tests on the PLATO units, given that interocular delays can influence stereopsis and luster (Ludwig et al., 2007). Unlike the CrystalEyes and nVidia systems, however, the liquid-crystal lenses of the PLATO glasses are highly transparent in the “open” state, making them much more amenable for naturalistic presentations of real-world objects. Our laboratory prototype glasses, like the PLATOs, have excellent transparency when the lenses are in the “open” state, thus making them superior to the CrystalEyes and nVidia systems for applications involving real-world solids. Another benefit of clear lenses is that the researcher can easily alternate between stimulus formats (i.e., real objects vs. 3-D stereo images) across consecutive trials.

Unlike the PLATO glasses, our prototype glasses were designed with a single panel of liquid-crystal material, rather than with two independently controlled lenses. In their current form, therefore, our prototype cannot be used for stereoscopic image presentations, as can the PLATO glasses or the other models described above. However, the prototype could, in principle, be modified to have a separate lens panel for each eye, each controlled by separate output circuits connected to the Arduino. Unlike the CrystalEyes and nVidia systems, there is no IR sensor to synchronize the lenses, but given the excellent onset/offset latencies revealed in our timing tests, the prototype may support stereoscopic presentations. Future iterations and developments of our prototype will be made available on our website (see Updates and future developments).

### Updates and future developments

Additional information and updates for users on how to manufacture liquid-crystal glasses hardware will be available on our Liquid-crystal Glasses Wiki (http://laboratorysys.com/glasses/). Useful additions could, over time, include (1) 3-D printable designs and materials for more comfortable glasses frames, or barrier setups; (2) new designs (and timing test results) for separately controlled left and right liquid-crystal lenses; (3) designs for accessories for use in high-field fMRI environments (such as liquid-crystal glasses without arms, or adjustable mounting devices for attaching the glasses to the head coil); (4) alternative or better-performing liquid-crystal materials; and (5) software updates. We will update the wiki periodically to accommodate future changes. Importantly, the Liquid-crystal Glasses Wiki allows users to contribute to the development of the hardware and software, which will ultimately facilitate and expand the application of liquid-crystal devices in vision research.

## Figures and Tables

**Table 1:** Descriptive statistics for the onset timing tests for the laboratory prototype (Lab Proto.), PLATO 1, and PLATO 2 glasses. Numerical values are milliseconds (msec)

Glasses	Side	Onset latency *Mean*	Onset latency *St. Dev.*	Min value	Max value	Mode	Frequency of the mode

Lab.Proto.	L	11.45	1.63	11	54	11	784
	R	11.52	1.55	11	47	11	773
	Mean	11.49	1.59				
PLATO 1	L	12.11	5.58	3	52	7	81
	R	15.26	6.19	5	73	16	70
	Mean	13.69	5.89				
PLATO 2	L	17.37	4.78	9	85	16	178
	R	16.89	4.07	10	53	16	177
	Mean	17.13	4.43				

**Table 2:** Statistical test results for the onset latency data

*Analysis of variance*		

Main effect	Statistic	*p*-value

Glasses	*F*(2,2997)= 760.63	*p* < 0.001*
Side	*F*(1,2997) = 75.09	*p* < 0.001*
Interaction		
Glasses × side	*F*(2,2997) = 114.64	*p* < 0.001*
*Post hoc paired-samples t-tests (two-tailed)*		
Left vs. right side		
Lab prototype	*t*(999) = 1.52	*p* = 0.13
Plato 1	*t*(999) = 12.40	*p* < 0.001*
Plato 2	*t*(999) = 2.62	*p* = 0.009*

* denotes statistically significant difference.

**Table 3:** Descriptive statistics for the offset timing tests for the Laboratory Prototype (Lab. Proto.), PLATO 1, and PLATO 2 glasses. Numerical values are milliseconds (msec)

Glasses	Side	Offset latency *Mean*	Offset latency *St. Dev.*	Min value	Max value	Mode	Frequency of the mode

Lab.Proto.	L	31.39	1.72	31	88	31	855
	R	31.40	1.64	31	89	31	858
	Mean	31.40	1.68				
PLATO 1	L	23.45	4.24	17	407	22	81
	R	24.90	5.51	16	82	20	70
	Mean	24.18	4.88				
PLATO 2	L	25.13	5.35	17	498	22	264
	R	19.64	4.21	14	432	18	143
	Mean	22.39	4.78				

**Table 4:** Statistical test results for the offset latency data.

*Analysis of variance*		

Main effect	Statistic	*p*-value

Glasses	*F*(2,2997)= 2,378.21	*p* < 0.001*
Side	*F*(1,2997)= 189.19	*p* < 0.001*
Interaction		
Glasses × side	*F*(2,2997)= 468.30	*p* < 0.001*
*Post hoc paired-samples t-tests (two-tailed)*		
Left vs. right side		
Lab prototype	*t*(999) = 0.23	*p* = 0.82
Plato 1	*t*(999) = 6.77	*p* < 0.001*
Plato 2	*t*(999) = 28.07	*p* < 0.001*

* denotes statistically significant difference.

## Data Availability

The data for our timing tests are publicly available at OSF (https://osf.io/ywms3).
